# Association of gut microbiota with idiopathic membranous nephropathy

**DOI:** 10.1186/s12882-022-02797-5

**Published:** 2022-04-30

**Authors:** Mengfei Li, Lixue Wei, Jing Sun, Qianshen Zhu, He Yang, Yu Zhang, Chao Zhang, Lin Xi, Rong Zhao, Xuanyi Du

**Affiliations:** 1 Department of Nephrology, the Second Affiliated Hospital of Harbin Medical University, 246 Xuefu Ave, Harbin, Heilongjiang, 150086 China; 2 Department of Nephrology, Jiaozuo People’s Hospital, 267 Jiefang Middle Road, Henan Jiaozuo, 454000 China

**Keywords:** Idiopathic membranous nephropathy, Gut microbiota, 16S rRNA sequencing

## Abstract

**Background:**

The prevalence of idiopathic membranous nephropathy (IMN) is increasing worldwide and the gut microbiota is recognized to play a role in its pathology. The aim of this study was to understand the involvement of the gut–kidney axis in IMN by analyzing the composition of the gut microbiota of biopsy-proven IMN patients compared with healthy controls (HC).

**Methods:**

Fecal samples from 30 patients with IMN diagnosed by renal biopsy and 30 healthy co-residents (control group) were collected for analysis in the Nephrology Department of the Second Affiliated Hospital of Harbin Medical University. The microbiota composition was analyzed by a 16S rRNA microbial profiling approach.

**Results:**

The results indicated that the α- and β-diversity of IMN patients differed significantly from those of the HC groups (*P* < 0.05). At the phylum level, IMN patients showed an increased abundance of Proteobacteria but a reduced abundance of Bacteroidota compared with the HC group. Actinobacteriota abundance showed a strong negative correlation with the estimated glomerular filtration rate. At the genus level, *Faecalibacterium*, *Agathobacter*, and *Bacteroides* were less abundant in the IMN group than in the HC group (LDA score > 2). Abundant bacterial functions related to lipid metabolism were observed among IMN group.

**Conclusion:**

Patients with IMN appear to have an altered gut microbiome, which could provide reference for future research on the interaction mechanism between the intestinal flora and IMN.

**Supplementary Information:**

The online version contains supplementary material available at 10.1186/s12882-022-02797-5.

## Introduction

Membranous nephropathy (MN), the main pathological type of adult nephrotic syndrome (NS), is an organ-specific autoimmune disease. The pathological characteristics of MN are diffuse deposition of glomerular basement membrane that forms subepithelial immune complexes, and diffuse thickening of the basement membrane [1]. According to the etiology, MN can be classified into idiopathic membranous nephropathy (IMN) and secondary membranous nephropathy (SMN). IMN is predominantly mediated by antibodies targeting the M-type phospholipase A2 receptor (anti-PLA2R) (85%) and thrombospondin type 1 domain containing 7A (anti-THSD7A) (3%–5%) [2]. Most of the autoantibodies from IMN patients belong to the IgG4 subclass [3]. IMN is characterized by heterogeneity of clinical outcomes, with 40% of patients undergoing spontaneous remission, and approximately 20% of patients progressing to severe kidney failure requiring renal replacement therapy [4]. The pathogenesis of MN is not yet clear, and may be related to environmental pollution [5], heredity, or autoimmunity.

The gut microbiome, residing in the intestine, is recognized as an important factor that contributes to both health and disease [6]. The composition of the intestinal flora is related to various diseases, such as inflammatory bowel disease [7,8], obesity [9], diabetes [10, 11], rheumatoid arthritis [12], atopic manifestations [13], liver sclerosis [14], cardiovascular disease [15], mental disease [16, 17], colorectal cancer [18, 19], and chronic kidney disease. The nonpodocyte circulating antigen, cationic bovine serum albumin, is believed to be the main antigen in early-childhood MN and may be affected by intestinal barrier formation and the use of infant formula [20]. Hence, we hypothesized that gut dysbiosis may play an important role in the development or exacerbation of IMN.

On the basis of next-generation sequencing technology, several human studies investigating the gut microbiota and IMN have been published recently [21–24]. Although all these studies indicated a compositional change in the fecal microbiota of IMN patients, their conclusions were not consistent. One possible explanation is that the participants were from different geographical locations and had different dietary habits, which may impact on gut microbial communities.

We thus conducted this study to investigate the fecal microbiota of IMN patients. We analyzed the bacterial community composition and diversity among patients and healthy individuals (cohabitants of the patients) using 16S rRNA gene sequencing. The role of important clinical parameters was also evaluated.

## Methods

### Study participants

A total of 30 hospitalized patients with IMN were enrolled in this study from May 2019 to December 2020 at the Second Affiliated Hospital of Harbin Medical University, along with 30 co-residents defined as healthy controls (HC) for whom urinary protein abnormalities had been ruled out. All participants, including patients and HC, were natives of Heilongjiang Province and were of Han ethnic group. The experimental group and the control group had similar dietary habits and living conditions.

The enrollment criteria for patients and their families were that they had not been administered antibiotics, probiotics, hormones, or immunosuppressants, and had not undergone any significant changes to diet or medication for at least 3 months. Exclusion criteria included diabetes, systemic lupus erythematosus, hepatitis B, hepatitis C, HIV**,** syphilis, and other diseases that can lead to SMN, as well as malignant tumors, gastrointestinal diseases, severe uncontrolled hypertension (diastolic blood pressure ≥ 120 mmHg and/or systolic blood pressure ≥ 220 mmHg), acute myocardial infarction or stroke within the last 6 months, and suspected/confirmed renal vascular disease. The demographic data and clinical features including sex, age, body mass index (BMI), estimated glomerular filtration rate (eGFR), total cholesterol (TC), triglyceride (TG), total protein (TP), alanine aminotransfease, aspartate aminotransferase, blood urea nitrogen, lactic dehydrogenase, high-density lipoprotein cholesterol (HDL-C), low-density lipoprotein (LDL-C), serum albumin were recorded.

This research received approval from the Ethics Committee from the Second Affiliated Hospital of Harbin Medical University in Harbin, Heilongjiang Province, China. All subjects provided written informed consent.

### Stool sample collection

Fresh fecal samples were collected from the participants in the morning. After collection, the samples were immediately frozen and stored at − 80 ℃ prior to analyses.

### DNA extraction and PCR amplification

Microbial community genomic DNA was extracted from fecal samples using the E.Z.N.A.® soil DNA Kit (Omega Bio-tek, Norcross, GA, USA) in accordance with the manufacturer’s instructions. The DNA extract was checked on a 1% agarose gel, and the DNA concentration and purity were determined using the NanoDrop 2000 UV–vis spectrophotometer (Thermo Fisher Scientific, Wilmington, MA, USA). The hypervariable region V3-V4 of the bacterial 16S rRNA gene was amplified with the primer pair 338F (ACTCCTACGGGAGGCAGCAG) and 806R (GGACTACHVGGGTATCTAAT) on an ABIGeneAmp®9700 PCR thermocycler (ABI, CA, USA). PCR amplification of the 16S rRNA gene was performed as follows: initial denaturation at 95 ℃ for 3 min, followed by 27 cycles of denaturing at 95 ℃ for 30 s, annealing at 55 ℃ for 30 s, and extension at 72 ℃ for 45 s, and a final extension step at 72 ℃ for 10 min and incubation at 10 ℃. The PCR mixture contained 4 µL of 5 × TransStartFastPfu buffer, 2 µL of 2.5 mM dNTPs, 0.8 µL of forward primer (5 µM), 0.8 µL of reverse primer (5 µM), 0.4 µL of TransStartFastPfu DNA polymerase, 10 ng of template DNA, and ddH_2_O up to a final volume of 20 µL. PCR reactions were performed in triplicate. The PCR product was then extracted from a 2% agarose gel and purified using the AxyPrep DNA Gel Extraction Kit (Axygen Biosciences, Union City, CA, USA) in accordance with the manufacturer’s instructions and quantified using the Quantus™ Fluorometer (Promega, USA).

### Illumina MiSeq sequencing

Purified amplicons were pooled in equimolar amounts and subjected to paired-end sequencing on an Illumina MiSeq PE300 platform/NovaSeqPE250 platform (Illumina, San Diego, CA, USA) following the standard protocols of Majorbio Bio-Pharm Technology Co. Ltd. (Shanghai, China).

### Processing of sequencing data

The raw 16S rRNA gene sequencing reads were demultiplexed, quality-filtered by fastp version 0.20.0, and merged by FLASH version 1.2.7 (https://ccb.jhu.edu/software/FLASH/index.shtml), in accordance with the following criteria: (i) the 300 bp reads were truncated at any site with an average quality score of < 20 over a 50 bp sliding window, and truncated reads shorter than 50 bp and reads containing ambiguous characters were discarded; (ii) only overlapping sequences longer than 10 bp were assembled according to their overlapping sequence. The maximum mismatch ratio of overlapping regions was 0.2. Reads that could not be assembled were discarded; and (iii) samples were distinguished according to their barcode and primers. The sequence direction was adjusted, barcodes had to be an exact match, and two nucleotide mismatches were permitted in primer matching.

Operational taxonomic units (OTUs) with a 97% similarity cutoff were clustered using UPARSE version 7.1, and chimeric sequences were identified and removed. The taxonomy of each OTU representative sequence was analyzed by RDP Classifier version 2.2 against the 16S rRNA database using a confidence threshold of 0.7. The current species annotation results are from the silva138 version(https://www.arb-silva.de/).

### Statistical analysis

Continuous data with or without a normal distribution were expressed as the mean ± SD or the median. Normally distributed data were assessed using Levene’s test to judge the homogeneity of the variance, and t-tests were performed for homogeneous variance. If the variance was non-uniform, Mann–Whitney U tests were used and counts were analyzed using chi-squared tests.

A rarefaction curve was plotted to evaluate the sufficiency of the sample size and to estimate bacterial richness. α-Diversity parameters, Sobs and Chao indices were used to estimate species richness while Shannon and Simpson’s indices were used to estimate species diversity, were analyzed by software mothur (version v.1.30.1). β-Diversity provides a comparison of the taxonomic profiles between pairs of individual samples. β-Diversity was calculated based on the Bray–Curtis distance matrices and displayed using principal coordinates analysis (PCoA) by the R software (version 3.3.1), and the analysis of differences between groups was tested by analysis of similarities (ANOSIM). Both α-diversity and β-diversity were calculated at the OTU level. Bacterial taxonomic comparisons at the phylum and genus levels were performed between two groups using the Wilcoxon rank sum test. Linear discriminant analysis (LDA) effect size (LEfSe) was used to identify the characteristic microbiota and explain the differences between patients and HC. Only taxa with an LDA score > 2 and a significance of α < 0.05 were shown. The results were plotted in a cladogram based on their phylogenetic relationship. The correlation between biochemical indicators and various microbes were calculated by Spearman’s rank correlation coefficient and visualized by heatmap in R using the pheatmap package. Relative predictive performance analysis was performed by random forest analysis in R using the randomForest package and receiver operating characteristic (ROC) curve analysis. The gut microbiota was explored based on Kyoto Encyclopedia of Genes and Genomes (KEGG, http://www.genome.jp/kegg/) using Phylogenetic Investigation of Communities by Reconstruction of Unobserved States (PICRUSt). Between-group differences in functional pathways indicated by taxa variations were assessed using the Mann–Whitney U test. *P* values < 0.05 were considered statistically significant. All statistical analyses were performed using the computer software Statistical package for the Social Sciences, version 25.0 (IBM Corp., Armonk, NY, USA).

## Results

### General characteristics of all participants

The study included 30 IMN patients, confirmed by renal biopsy, with a mean age of 51.3 ± 10.25 years. All IMN patients had an estimated glomerular filtration rate(eGFR) ≥ 55 mL/min/1.73 m^2^. The baseline characteristics of the IMN and control groups are summarized. (Table 1, Supplemental Table 1).

**Table 1:** Baseline characteristics of participants

**Variables**	**IMN (*n* = 30)**	**HC (*n* = 30)**	***P* value**
Age (years)	51.30 ± 10.25	45.20 ± 13.24	0.051
Gender (female/male)	8 / 22	15 / 15	0.11
BMI (kg/m^2^)	21.89 ± 1.55	21.26 ± 1.30	0.094
eGFR (ml/min/1.73m^2^)	88.85 ± 22.19		
Urinary protein excretion (g/24 h)	5.40 ± 3.67		
TP (g/L)	52.43 ± 8.83		
ALB (g/L)	26.12 ± 6.77		
TC (mmol/L)	7.88 ± 1.99		
TG (mmol/L)	3.45 ± 3.29		
HDL-C (mmol/L)	1.25 ± 0.27		
LDL-C (mmol/L)	5.17 ± 1.44		
SBP (mmHg)	138 ± 17		
DBP (mmHg)	90 ± 11		
UA (umol/L)	373.71 ± 113.29		
ALT (U/L)	18.1 ± 31.05		
AST (U/L)	24 ± 24.83		
BUN (mmol/L)	5.81 ± 2.34		
LDH (U/L)	223.08 ± 51.36		

*Note*: Results are expressed as the mean ± SD and ratio

*Abbreviations*: *SD* Standard deviation, *BMI* Body mass index, eGFR Estimated glomerular filtration rate, *TC* Total cholesterol, *TG* Triglyceride, *TP* Total protein, *ALT* Alanine aminotransferase, *AST* Aspartate aminotransferase, *BUN* Blood urea nitrogen, *LDH* Lactic dehydrogenase, *HDL-C* High-density lipoprotein cholesterol, *LDL-C* low-density lipoprotein, *ALB* serum albumin

### Intestinal flora diversity

A total of 2,075,757,404 reads were obtained for the 60 subjects by V4V5 16S rRNA pyrosequencing. After quality control and paired-end read merging, we obtained 1,418,592,102 high-quality reads, accounting for 68.34% of the total reads. A total of 3,448,102 sequences for all samples were used in downstream bioinformatics analysis, and the average sequence length of the merged sequences was 412 bp.

**Fig. 1:**
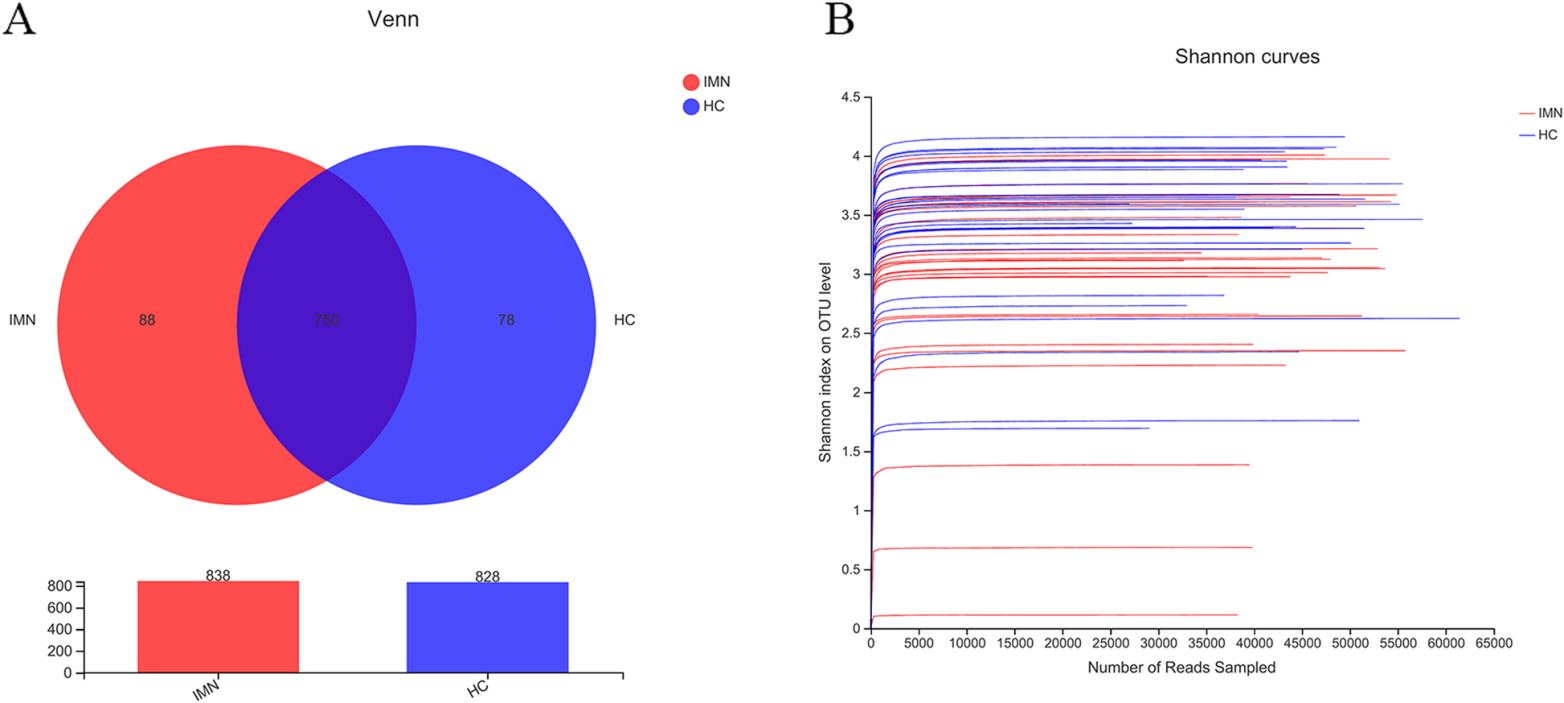
Gut microbiota compositions differed between IMN and HC groups. **A** Venn diagram of IMN and HC; **B** rarefaction analysis of both groups.The curves in all samples were near saturation, which means the sequencing depth was sufficient to capture most gene diversity

A total of 916 OTUs were obtained at a 97% homology level cutoff. The number of OTUs was larger for the HC group. As exhibited in the Venn diagram (Fig. 1A), the number of common OTUs between the HC and IMN groups was 750; the IMN group had 88 specific OTUs and the HC group had 78 specific OTUs not shared by the other group.

**Fig. 2:**
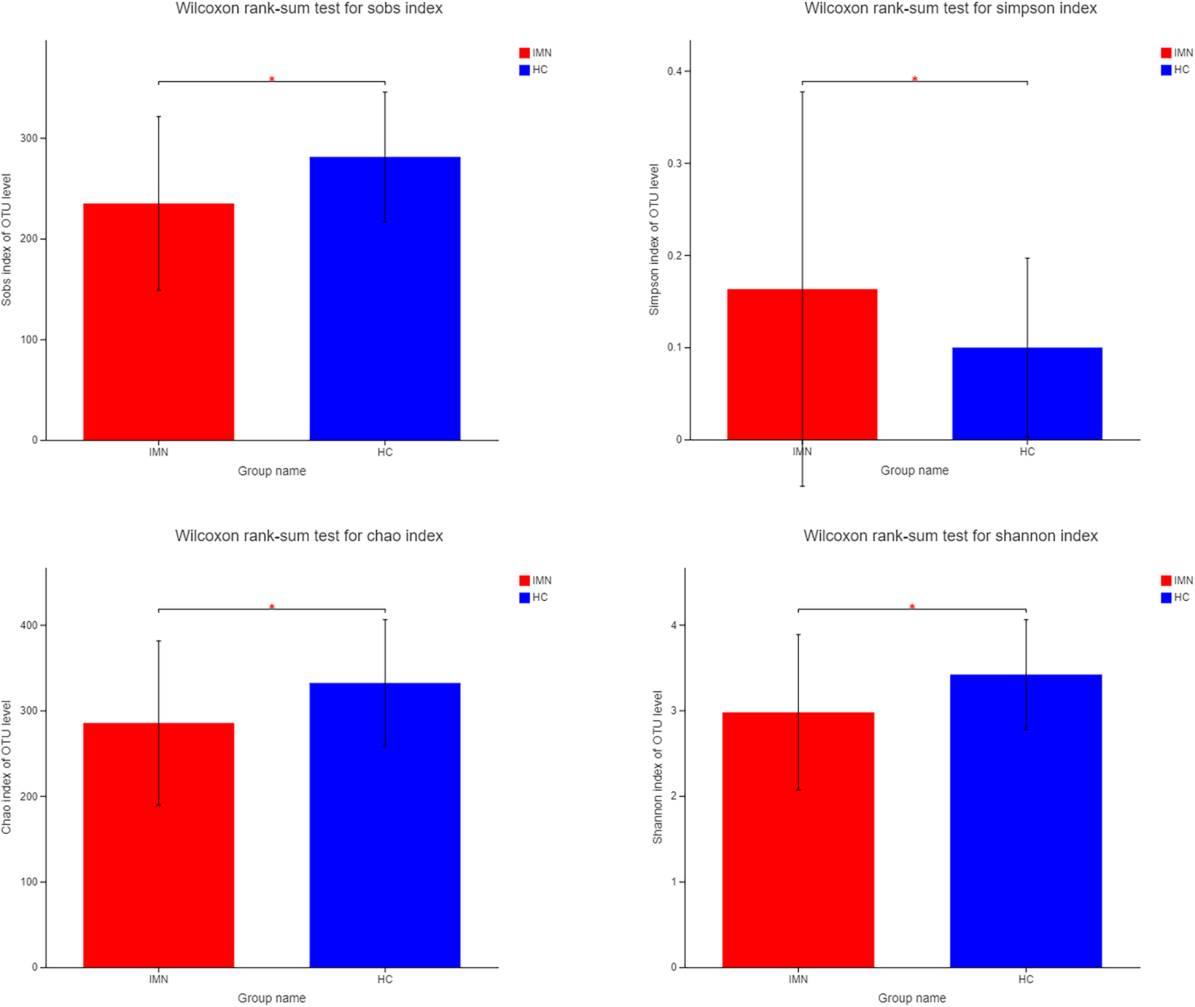
Composition of gut microbiota is significantly altered in primary hypothyroidism patients. α-Diversity indices (Chao, Shannon, Simpson and Sobs index) of intestinal flora in healthy individuals and Idiopathic membranous nephropathy patients. The Wilcoxon rank sum test was used to detect significant changes. *P < 0.05; **P < 0.01. HC: healthy individuals, *n* = 30; IMN: Idiopathic membranous nephropathy, *n* = 30

To determine whether the sequencing adequately captured the diversity of the gut microbiota, rarefaction analysis was performed. The curves in all samples were near saturation, which meant that the sequencing depth was sufficient to capture most of the gene diversity (Fig. 1B). Chao and Sobs indices on the OTU profile were used to estimate community richness. Shannon and Simpson indices were used to estimate community diversity. α-Diversity analysis revealed significant differences among groups; i.e., there was a significantly lower diversity in IMN patients compared with the HC group (Chao, *P* = 0.048; Sobs, *P* = 0.024; Shannon, *P* = 0.016; Simpson, *P* = 0.032) (Table 2, Fig. 2, Supplemental Table 2).

**Table 2:** α-Diversity between HC and IMN

Estimators	HC-Mean	HC-SD	IMN-Mean	IMN-SD	*P* value	*Q* value
Sobs	281.03	64.458	234.93	86.261	0.02415	0.05442
Shannon	3.4188	0.64087	2.9768	0.90888	0.01695	0.05442
Simpson	0.099691	0.097096	0.16319	0.21393	0.03265	0.05442
Chao	332.07	74.061	285.32	95.84	0.04841	0.05555

*Abbreviations*: *HC* Health control, *IMN* Idiopathic membranous nephropathy

**Fig. 3:**
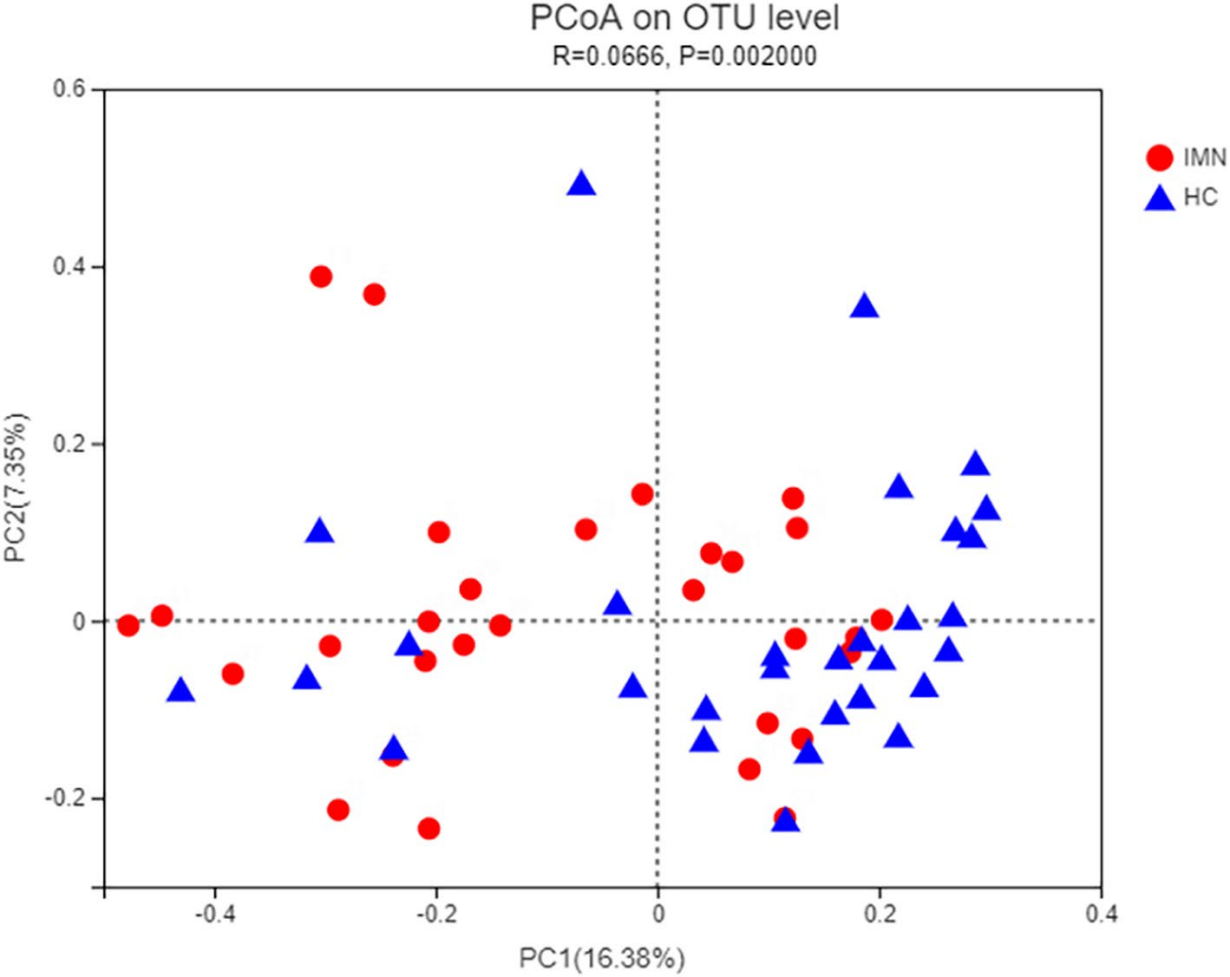
A principal component (PCoA) score plot based on Bray–Curtis distance matrix for all participants. Each point represents the composition of the intestinal microbiota of one participant. The ellipses do not represent statistical significance but rather serve as a visual guide to illustrate group differences. The ANOSIM test was used

Principal co-ordinates analysis (PCoA) searches for main coordinates based on a distance matrix; i.e., shows an higher similarity when OTUs decreased. PCoA based on Bray–Curtis dissimilarity at the OTU level revealed that the microbiota composition of IMN and HC patients was significantly different (*P* = 0.002, *r* = 0.066) (Fig. 3).

### Bacterial taxa differences between IMN patients and healthy controls

**Fig. 4:**
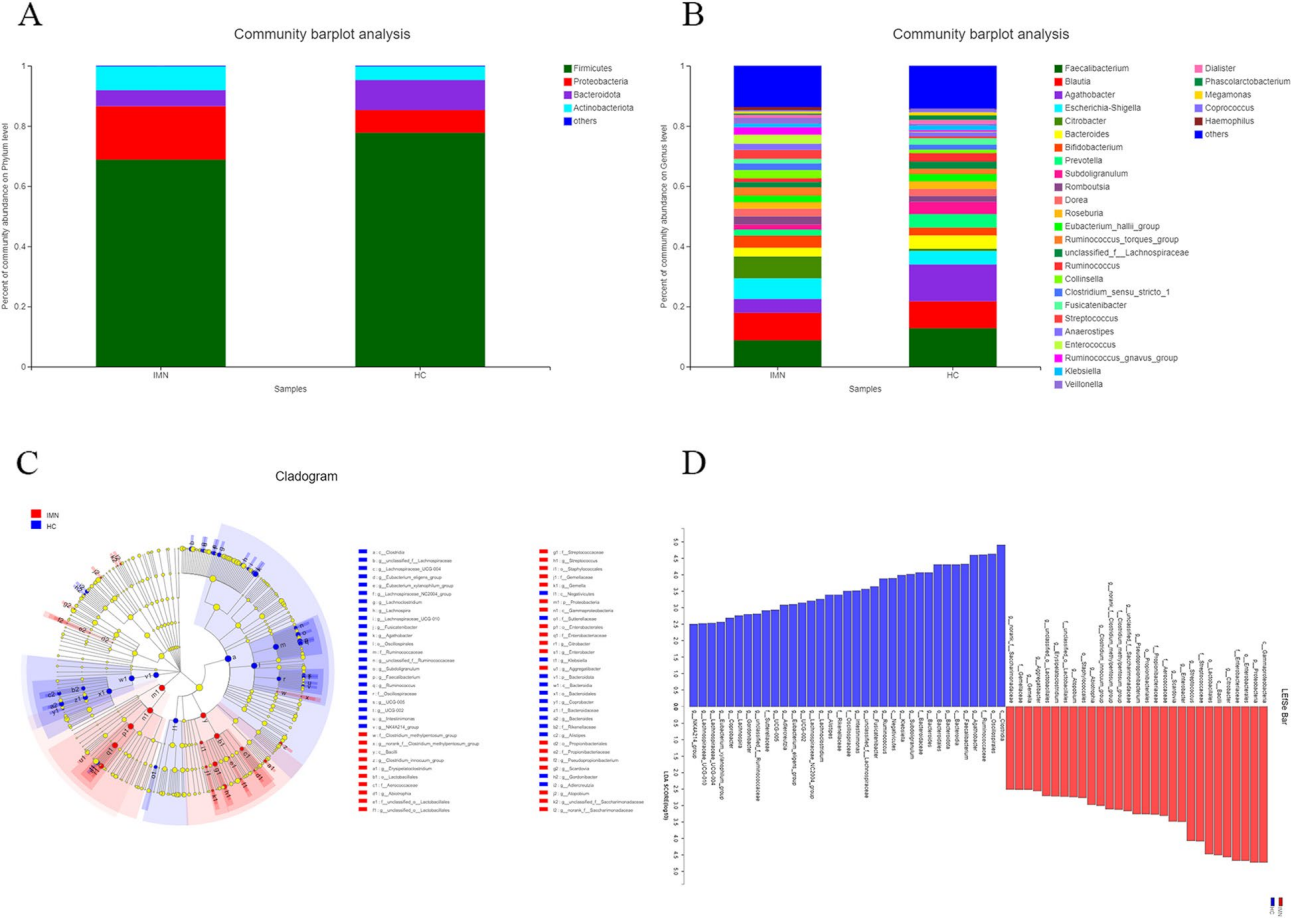
Relative abundance and taxonomic differences of fecal microbiota in the IMN patients and healthy controls. **A** Microbiome composition in the two groups at the phylum level. **B** Microbiome composition in the two groups at the genus level. The figure shows the top 20 species in each group based on their relative abundances. **C**, **D** LDA and LEfSe of IMN and HC

Both IMN patients and HC showed typical microbiome structures. Most bacteria identified belonged to the phyla Bacteroidota, Firmicutes, Proteobacteria, and Actinobacteriota. IMN patients showed an increased abundance of Proteobacteria and Actinobacteriota and a decreased abundance of Bacteroidota and Firmicutes (Fig. 4A).

Within the Proteobacteria, the abundance of Enterobacteriaceae was significantly higher in IMN patients than in the HC at the family level (*P* < 0.05). For the Bacteroidota family, a significant decrease was found in the abundance of Bacteroidaceae in the IMN group compared with the HC group (*P* < 0.01) (Table 3).

**Table 3:** Relative abundance of fecal microbiota in IMN patients and healthy controls

	**HC-Mean(%)**	**HC-SD(%)**	**IMN-Mean(%)**	**IMN-SD(%)**	***P* value**
**Phylum level**					
p__Proteobacteria	8.92	16.03	18.68	22.8	0.01501
p__Bacteroidota	10.3	15.74	5.295	9.608	0.01327
p__Patescibacteria	0.02846	0.03447	0.04899	0.04299	0.03776
p__unclassified_k__norank_d__Bacteria	0.0509	0.1048	0.01423	0.02254	0.005974
p__Desulfobacterota	0.03305	0.0641	0.01276	0.03438	0.005956
**Family level**					
f__Ruminococcaceae	19.44	11.62	11.11	10.83	0.005084
f__Enterobacteriaceae	8.49	15.97	17.07	23.13	0.02068
f__Bacteroidaceae	4.809	5.384	3.012	7.338	0.008315
f__Streptococcaceae	0.6491	0.9291	2.929	6.587	0.004637
f__Oscillospiraceae	1.086	1.269	0.4444	0.6904	0.00172
f__Rikenellaceae	0.6509	0.9883	0.1596	0.3023	0.005897
**Genus level**					
g__Faecalibacterium	11.98	9.315	7.973	9.573	0.04594
g__Agathobacter	11.86	13.66	4.344	7.221	0.001904
g__Citrobacter	0.8159	2.388	7.623	18.29	0.02412
g__Bacteroides	4.809	5.384	3.012	7.338	0.008315
g__Subdoligranulum	3.879	3.348	1.612	2.058	0.002985
g__unclassified_f__Lachnospiraceae	2.357	1.082	1.753	1.172	0.02068
g__Ruminococcus	2.813	3.237	1.154	1.355	0.004511
g__Fusicatenibacter	2.091	1.722	1.632	3.644	0.004123
g__Streptococcus	0.6226	0.9319	2.836	6.576	0.008315
g__Klebsiella	2.235	11.06	1.181	0.04594	0.01425
g__Lachnoclostridium	0.8989	1.101	0.7211	1.28	0.01765
g__Enterobacter	0.3569	1.616	0.8472	1.887	0.02282
g__Alistipes	0.6498	0.9881	0.1596	0.3023	0.006454
g__Lachnospiraceae_NC2004_group	0.5385	0.4596	0.2454	0.3116	0.005287
g__Adlercreutzia	0.4476	0.6586	0.2314	0.614	0.01156
g__UCG-002	0.4722	0.6633	0.202	0.3951	0.01068
g__unclassified_f__Ruminococcaceae	0.261	0.2816	0.1273	0.1812	0.007382
g__UCG-005	0.2622	0.4632	0.08283	0.1824	0.008302
g__Eubacterium_eligens_group	0.2999	0.792	0.04314	0.1003	0.022
g__Lachnospira	0.1659	0.3743	0.0685	0.1373	0.002307
g__Clostridium_innocuum_group	0.02968	0.1065	0.1943	0.6849	0.04614
g__Erysipelatoclostridium	0.03047	0.1001	0.123	0.3534	0.0007543

*P* < 0.05 is statistically significant

At the genus level, *Bacteroides, Faecalibacterium, Escherichia-Shigella, Agathobacter*, and *Citrobacter* were the most abundant genera in both groups. Some of these genera showed a significant difference between these two groups (Fig. 4B, Table 3).

To determine different taxa from the phylum to genus level among the two groups, the LEfSe algorithm was used. Bacterial species that differed significantly (*P* < 0.05) between the IMN patients and HC were screened using LEfSe to construct a cladogram (Fig. 4C, D).

The histogram obtained by LDA showed the increased abundances of 19 genera in the IMN group. Among the HC group, the abundances of 29 genera were significantly higher than for the IMN patients. (Supplemental Table 6).

### Association between the fecal microbiota and IMN clinical characteristics

**Fig. 5:**
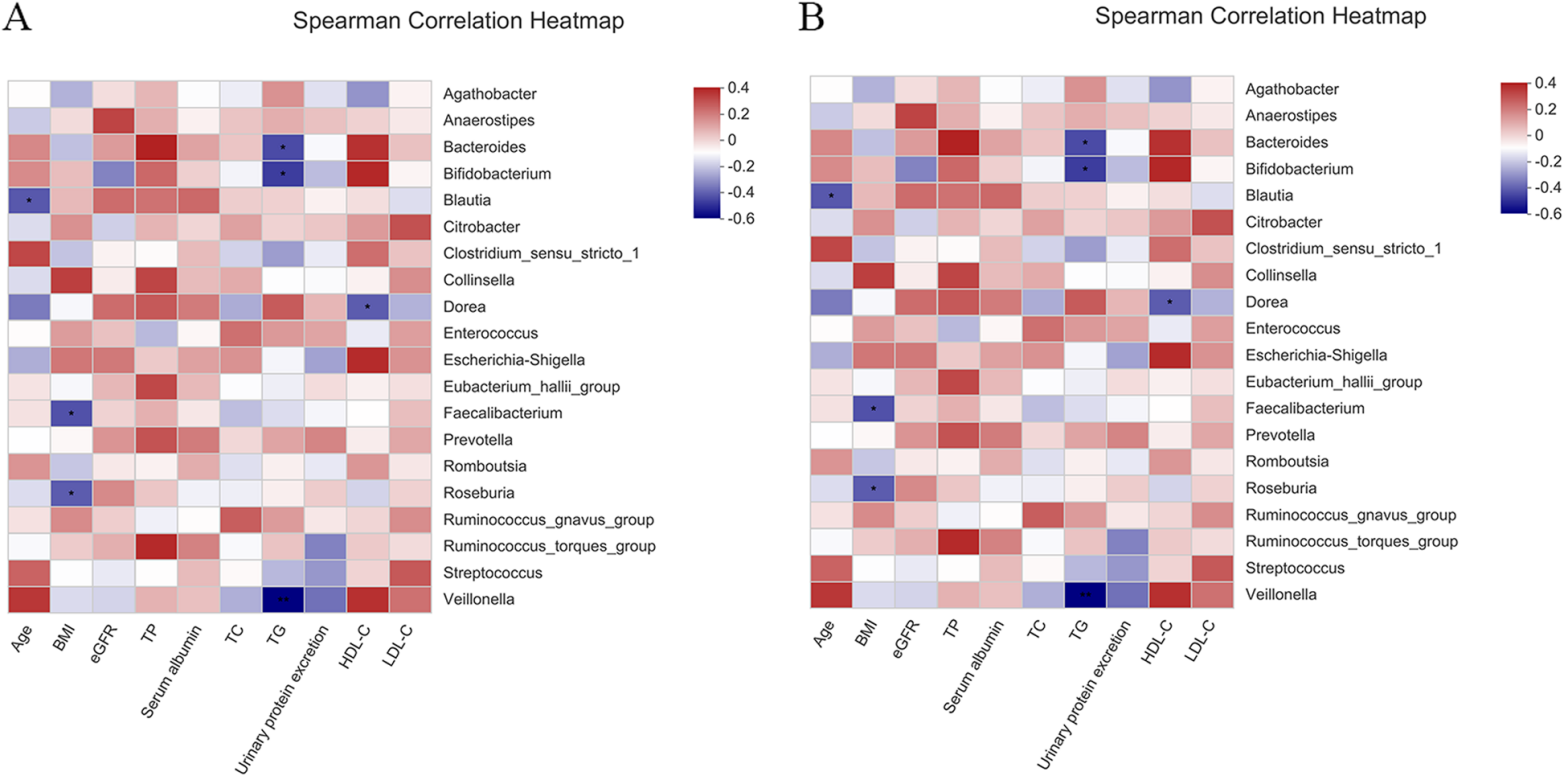
Heatmaps showing correlations between fecal microbiota and IgAN clinical parameters. **A** Correlations between microbiota phyla and IMN clinical characteristics. **B** Correlations between microbiota genera and IMN clinical characteristics. The correlations were performed by heatmap in R version 3.3.1 (https://cran.r-project.org/) using the pheatmap package. The figure shows the top 20 species in each group based The intensity of the color indicates the r value (correlation). The red color represent positive score and the blue color represent negative score. *P < 0.05 and **P < 0.01

Correlations between the differences in gut microbiota and clinical biomarkers were further evaluated by Spearman’s rank correlation analysis. At the phylum level, Actinobacterioia showed a strong negative correlation with the eGFR (*r* =  − 0.414, *P* = 0.023), while Campylobacterota showed a positive correlation with LDL-C (*r* = 0.375, *P* = 0.041) and Bacteroidota showed a positive correlation with TP (*r* = 0.373, *P* = 0.043) (Fig. 5A, Supplemental Table 3).

At the genus level, *Bacteroides*, *Bifidobacterium*, and *Veillonella* showed strong negative correlations with TG (*r* =  − 0.420, *P* = 0.021; r =  − 0.447, *P* = 0.013; − 0.555, *P* = 0.001). *Dorea* also showed a negative correlation with HDL-C (*r* =  − 0.390, *P* = 0.033). *Faecalibacterium* and *Roseburia* exhibited a negative correlation with BMI (*r* =  − 0.409, *P* = 0.024; *r* =  − 0.390, *P* = 0.033). A negative correlation also existed between *Blautia* and age (*r* =  − 0.397, *P* = 0.030) (Fig. 5B, Supplemental Table 4).

### Microbial markers for the potential detection of IMN

**Fig. 6:**
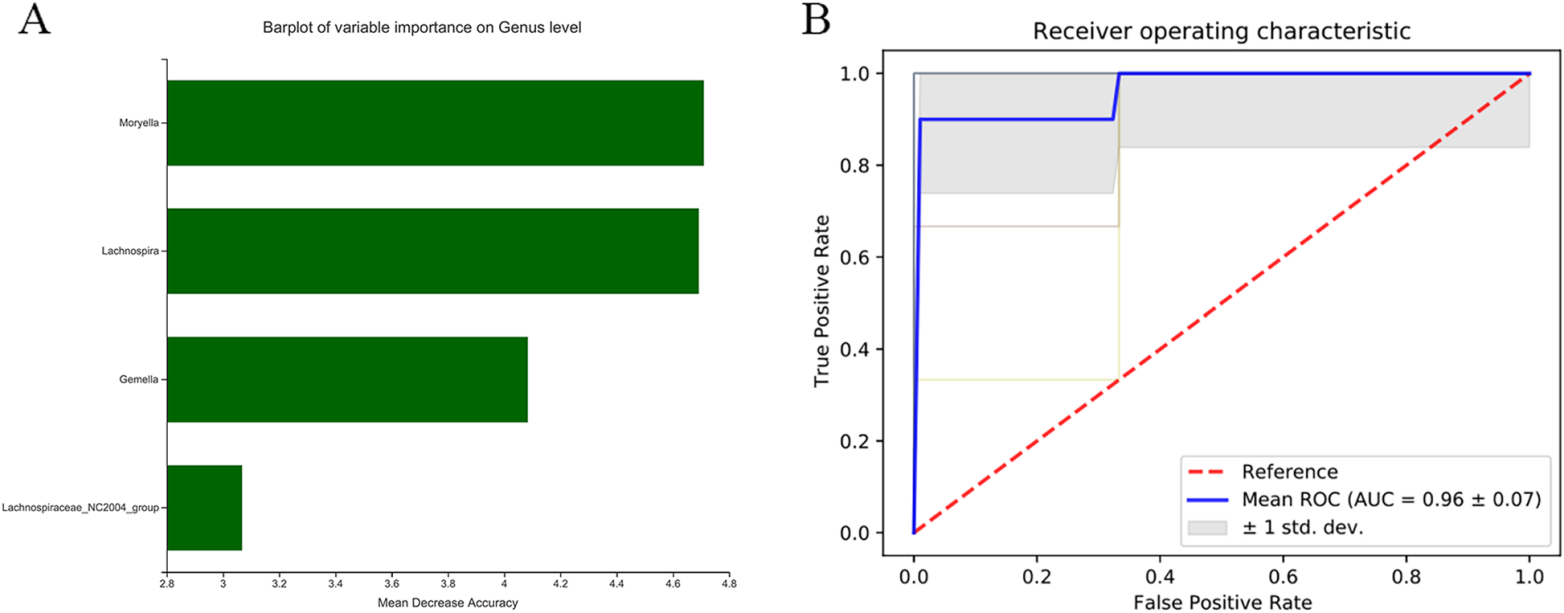
The predictive model based on the relative abundances at genus level by using RF model. **A** The importance of each genus in the predictive model was evaluated by the mean decreasing accuracy and the Gini coefficient. **B** ROC curve analysis generated by the RF using 4 genera in the fecal microbiota

Four bacteria, determined by random forest analysis, were found to be characteristic of IMN (Fig. 6A). To illustrate the microbial signatures of IMN patients and HC and to further build a predictive model according to the fecal microbiota profiles of the significantly different taxa abundances at the genus level, ROC curves for classifying IMN from HC were developed. We could detect IMN individuals accurately based on the combination of the four genera (*g__Moryella, g__Lachnospira, g__Gemella, g__Lachnospiraceae_NC200 4_group*), as indicated by the area under the receiver operating curve (AUC) of up to 0.96 (Fig. 6B).

### Functional pathway predictions

**Fig. 7:**
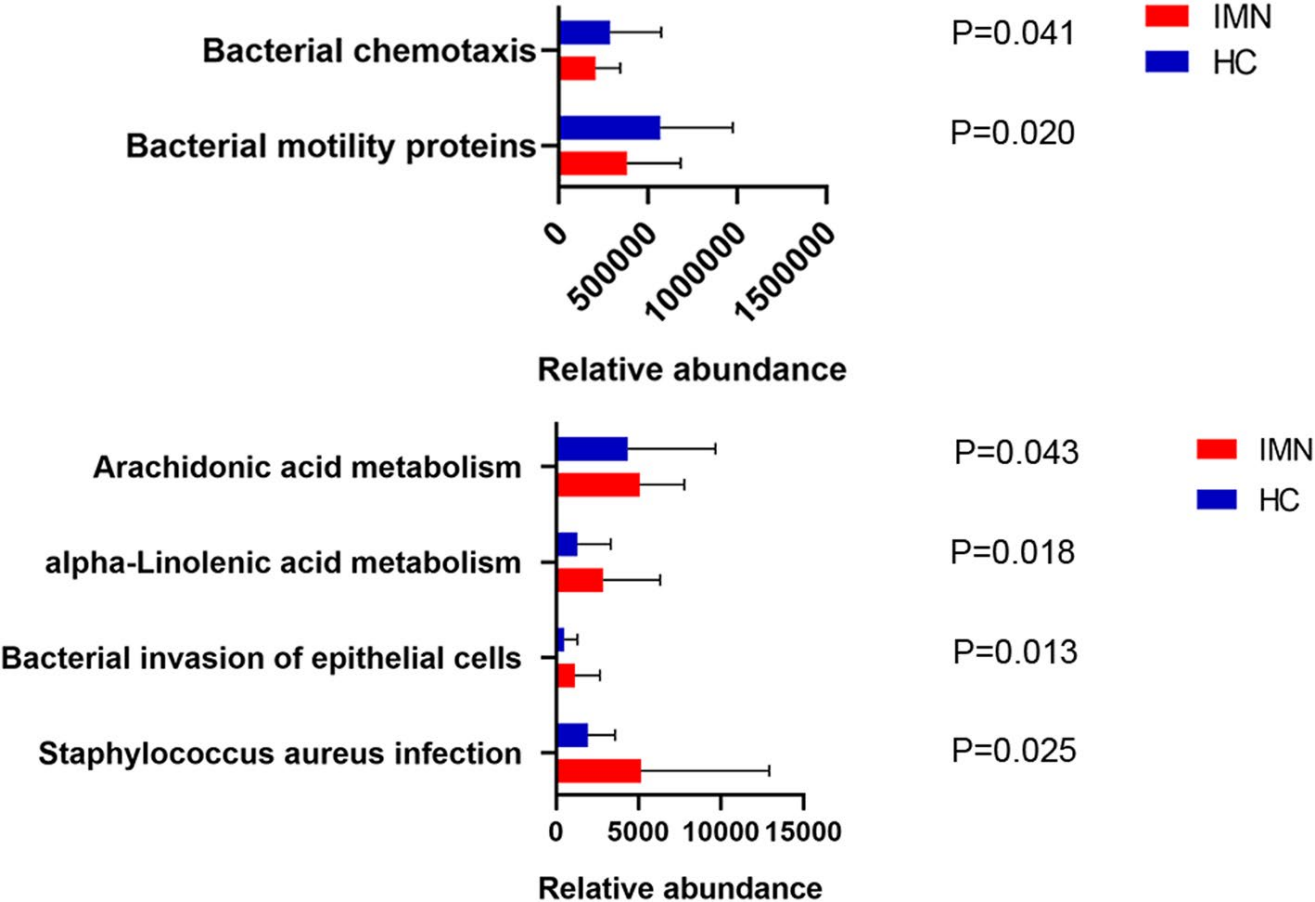
Predicted microbial functions using PICRUSt. Significant KEGG pathways (http://www.kegg.jp/kegg/kegg1.html) at level 3 between the IMN patients and healthy controls. *P* < 0.05 is statistically significant

From metagenome predictions based on PICRUSt analysis, abundant bacterial functions related to lipid metabolism were observed among IMN cases and the controls. At the level of the KEGG pathway, microbial gene functions included pathways involved in bacterial invasion of epithelial cells (ko05100), Alpha-linolenic acid metabolism (ko00592), Staphylococcus aureus infection (ko05150), and Arachidonic acid metabolism (ko00590), which were higher in the fecal microbiome of the IMN group compared with the control group (*P* < 0.05; Fig. 7, Supplemental Table 5). This indicated the differential function of the fecal microbiota between IMN patients and healthy individuals.

## Discussion

The intestinal microecosystem is continuously evolving, and its microbial diversity and abundance play important roles in maintaining a normal physiological state; however, the host also influences the intestinal microflora [25]. In this study, 16S rRNA sequencing was performed on stool samples from IMN patients and healthy co-residents. The relative abundances of each intestinal microbial species and their diversities and compositions were compared between IMN patients and HC. The results showed significantly decreased intestinal flora diversity and an altered intestinal flora composition in the IMN patients compared with those of the HC.

External factors (such as changes in diet or geographical region) and internal factors (such as physiological changes in response to a disease process) can both induce shifts in microbiota composition and function [26, 27]. We recruited the patients co-residents as the control group to reduce the influence of diet and living conditions on the results. Our findings demonstrated that the community richness of the fecal microbiota in the IMN patients was significantly lower than that in the HC, which was consistent with previous observations [21–24]. However, there were differences in the taxonomic profile of the fecal microbiota compared with previous research.

Bacteroidota, Firmicutes, Proteobacteria, and Actinobacteriota were the predominant phyla in both groups, which was consistent with reports from previous Chinese studies [21]. When compared with the HC, Firmicutes and Bacteroidota were depleted in patients with IMN. The phylum Bacteroidota produces acetate and propionate, whereas Firmicutes produces butyrate as its primary metabolic end product [28]. Proteobacteria not only possesses genes that encode the urease enzyme [29] but also promotes inflammatory uremic toxins [30].

Proteobacteria is regulated by a gamma-Proteobacteria-specific IgA response, thus a higher abundance of gamma-Proteobacteria promotes the production of inflammation [31]. Proteobacteria and Fusobacteria have also been found to be enriched in type 2 diabetic patients with chronic kidney disease [32].

The main genera that differed between the IMN and HC groups were *Agathobacter*, *Faecalibacterium*, *Bacteroides*, and *Subdoligranulum*. However, the physiological and pathological effects of these genera have not been investigated in detail. *Bacteroides* is a dominant intestinal organism that plays a pivotal role in improving nutrient utilization, accelerating intestinal mucosal angiogenesis, developing the immune system, improving immunity, and maintaining the balance of intestinal flora. *Bacteroides* can produce short-chain fatty acids in the colon, and significantly enhance the growth of colonic lamina propria Treg cells after colonization. Such enhancement further exerts immunomodulatory effects [22]. *Faecalibacterium* is abundant in the fecal microbiome of healthy humans. It is a member of Clostridium IV (Firmicutes) and produces short-chain fatty acids (including butyrate) [33].

Collectively, IMN correlates with changes in abundance of some bacterial taxa. Spearman correlation analysis confirmed that opportunistic pathogens, such as Actinobacteriota, negatively correlated with the eGFR, which is classical marker of renal damage. Patients with IMN often exhibit hyperlipidemia. *Dorea*, which has been shown to be closely related to obesity [34], was negatively correlated with HDL-C levels and was significantly increased in IMN patients. *Bacteroides*, *Bifidobacterium*, and *Veillonella*, associated with adiposity and lipid levels [35], were negatively correlated with TG in our research. PICRUSt analysis indicated that the gut microbiota that were altered in abundance between IMN patients and HC were related to lipid metabolism. In 1982, Moorhead [36] proposed the hypothesis that lipids exert renal toxicity. In recent years, research has shown that abnormal blood lipid levels can cause renal glomerular membrane hyperplasia, epidural matrix aggregation, renal glomerular epithelial cell damage, and inflammatory cell immersion, all of which can damage the kidney directly [37]. Hyperlipidemia, defined as one of the characteristics of IMN, increases blood viscosity and the probability of venous thrombosis, and accelerates the progression of renal damage.

The results of our study demonstrated that IMN could be distinguished relatively accurately from HC via the detection of four specific genera in feces. However, further studies are required to establish the exact role of the gut microbiota in IMN progression and to identify diagnostic and predictive markers.

Current treatments, including supportive therapy, corticosteroids and immunosuppressive agents, are not effective in all patients. In one reported case, fecal microbiota transplantation as used to treat a patient with MN and chronic diarrhea, whose symptoms ameliorated and renal function improved [38]. Research indicates that the gut microbiota may contribute to the pathogenesis of this disease.

Our study had certain limitations. First, 16S rRNA sequencing was performed among participants from the same area of residence (Han nationality). Second, our sample size was relatively small; therefore, a large-scale study involving different populations and strict dietary controls are needed to confirm our results. We minimized the influence of other factors, such as weight, BMI, sex, and diet in our study; however, the proportion of certain ingredients in the diet (such as salt or fatty acids) was not controlled. Third, to identify further novel microbial and metabolic biomarkers and to further characterize the biological mechanisms and pathways involved, bacterial metagenomic sequencing and metabonomic analysis are needed in future studies. Finally, a cross-sectional study cannot confirm a causal relationship because of variations in the gut microbiota over time.

Overall, the up- or down-regulation of these strains may be closely related to the occurrence of IMN. The molecular mechanisms involved in the relationship between IMN and intestinal bacterial changes will be studied in future analyses.

## Conclusion

By comparing the intestinal flora of IMN and HC, we found that microbial diversity was significantly different in IMN patients. The variations identified in the gut microbiome will provide a preliminary reference for future research on the interaction mechanism between the intestinal flora and IMN.

## Supplementary Information

**Additional file 1: Supplemental Table 1. **Baseline characteristics of participants.

**Additional file 2: Supplemental Table 2. **α-Diversity was assessed with 4 different diversity analysis methods.

**Additional file 3: Supplemental Table 3. **Gut microbiota and clinical biomarkers evaluated by Spearson correlation analysis at the phylum level.

**Additional file 4: Supplemental Table 4. **Gut microbiota and clinical biomarkers evaluated by Spearson correlation analysis at the genus level.

**Additional file 5: Supplemental Table 5. **KEGG level 3 pathways of abundance changed intestinal flora.

**Additional file 6: Supplemental Table 6. **Bacterial species that differed significantly between the IMN patients and HC by LEfSe.

## Data Availability

All data generated or analyzed during this study are included in this published article.

## Abbreviations

ANOSIM: Analysis of similarities
AUC: Area under curve
BMI: Body mass index
eGFR: Estimated glomerular filtration rate
ESRD: End-stage renal disease
FMT: Fecal microbiota transplantation
HDL-C: High density liptein cholesterol
IgAN: Immunoglobulin A nephropathy
IMN: Idiopathic membranous nephropathy
KEGG: Kyoto Encyclopedia of Genes and Genomes
LDL-C: Low-Density Lipoprotein Cholesterol
LEfSe: Linear discriminant analysis effect size
OTUs: Operational taxonomic units
PCoA: Principal coordinates analysis
PICRUSt: Phylogenetic Investigation of Communities by Reconstruction of Unobserved States
RF: Random forest
SMN: Secondary membranous nephropathy
TC: Serum total cholesterol
TG: Triglyceride
TP: Total protein
